# Direct In Vivo Activation of T Cells with Nanosized Immunofilaments Inhibits Tumor Growth and Metastasis

**DOI:** 10.1021/acsnano.2c11884

**Published:** 2023-06-20

**Authors:** Lea Weiss, Jorieke Weiden, Yusuf Dölen, Emilia M. Grad, Eric A. W. van Dinther, Marjolein Schluck, Loek J. Eggermont, Guido van Mierlo, Uzi Gileadi, Ariadna Bartoló-Ibars, René Raavé, Mark A. J. Gorris, Lisa Maassen, Kiek Verrijp, Michael Valente, Bart Deplancke, Martijn Verdoes, Daniel Benitez-Ribas, Sandra Heskamp, Annemiek B. van Spriel, Carl G. Figdor, Roel Hammink

**Affiliations:** 1 Department of Tumor Immunology, Radboud Institute for Molecular Life Sciences, Radboud University Medical Center, Geert Grooteplein 26, 6525 GA Nijmegen, The Netherlands; 2 Division of Immunotherapy, Oncode Institute, Radboud University Medical Center, 6525 GA Nijmegen, The Netherlands; 3 Institute for Chemical Immunology, 6525 GA Nijmegen, The Netherlands; 4 Laboratory of Systems Biology and Genetics, Institute of Bioengineering, School of Life Sciences, Swiss Federal Institute of Technology (EPFL), 1015 CH Lausanne, Switzerland; 5 MRC Human Immunology Unit, Weatherall Institute of Molecular Medicine, University of Oxford, OX3 9DS Oxford, United Kingdom; 6 Department of Immunology, Hospital Clinic, August Pi I Sunyer Biomedical Research Institute (IDIBAPS), University of Barcelona, Carrer Villarroel 170, 08036 Barcelona, Spain; 7 Department of Radiology and Nuclear Medicine, Radboud University Medical Center, Geert Grooteplein-Zuid 10, 6525 HP Nijmegen, The Netherlands

**Keywords:** immunotherapy, immunofilaments, antigen-specific T cells, T cell expansion, CAR T cells, antitumor activity

## Abstract

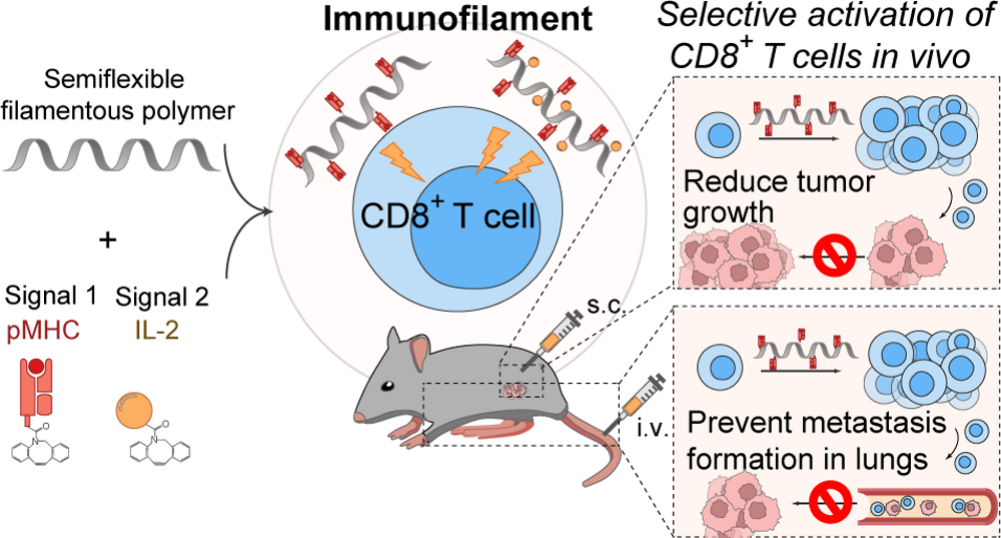

Adoptive T cell therapy has successfully been implemented for the treatment of cancer. Nevertheless, ex vivo expansion of T cells by artificial antigen-presenting cells (aAPCs) remains cumbersome and can compromise T cell functionality, thereby limiting their therapeutic potential. We propose a radically different approach aimed at direct expansion of T cells in vivo, thereby omitting the need for large-scale ex vivo T cell production. We engineered nanosized immunofilaments (IFs), with a soluble semiflexible polyisocyanopeptide backbone that presents peptide-loaded major histocompatibility complexes and costimulatory molecules multivalently. IFs readily activated and expanded antigen-specific T cells like natural APCs, as evidenced by transcriptomic analyses of T cells. Upon intravenous injection, IFs reach the spleen and lymph nodes and induce antigen-specific T cell responses in vivo. Moreover, IFs display strong antitumor efficacy resulting in inhibition of the formation of melanoma metastases and reduction of primary tumor growth in synergy with immune checkpoint blockade. In conclusion, nanosized IFs represent a powerful modular platform for direct activation and expansion of antigen-specific T cells in vivo, which can greatly contribute to cancer immunotherapy.

To date, the cancer immunotherapy field is dominated by therapeutic strategies that aim to exploit the cytotoxic potential of T cells. T cells can trigger tumor cell death following the recognition of tumor antigens presented as peptide epitopes on major histocompatibility complexes (pMHC) on the cell surface. In particular, unleashing existing tumor-directed T cell responses by administrating monoclonal antibodies that block coinhibitory receptors PD-1 and/or CTLA-4 on T cells has proven highly beneficial, and is termed immune checkpoint blockade.^1−4^ To further enhance the proportion of cancer patients that respond to immunotherapy, these strategies are complemented with treatment modalities aimed at expanding the number of already existing T cells or inducing novel tumor-specific T cells. To this end, cancer patients are treated with adoptive T cell therapy (ACT), in which they receive infusions of ex vivo expanded tumor-infiltrating lymphocytes (TILs) or genetically engineered chimeric antigen receptor (CAR)-expressing T cells.

Artificial antigen-presenting cells (aAPCs) provide an essential off-the-shelf tool for the ex vivo expansion of T cells for ACT. These aAPCs mimic the interaction between natural APCs such as dendritic cells (DCs) and T cells during T cell priming. To this end, synthetic constructs have been designed that present molecular signal to T cells in a controlled manner to (1) trigger T cell receptor (TCR) signaling by presenting agonistic anti-(α)CD3 antibodies or antigen-specific pMHC, and (2) provide secondary signals by triggering costimulatory receptors such as CD28 to enhance and prolong T cell responses. In addition, cytokines such as interleukin-2 (IL-2) or type I interferons can be provided as a third signal to promote T cell survival, control T cell phenotype, and enhance T cell functionality. The most notable example of aAPCs that are applied in clinical settings are micrometer-sized iron oxide particles (Dynabeads), which typically present αCD3/αCD28 antibodies to foster ex vivo polyclonal T cell expansion. Various alternative synthetic systems have been developed that support more rapid T cell expansion or promote a favorable T cell functionality or phenotype.^5−9^ However, large-scale ex vivo multiplication of T cells remains laborious and costly, and can also compromise T cell functionality and viability, leading to a suboptimal therapeutic efficacy for current ACT strategies.^10−13^ Here, we propose a radically different approach by designing nanosized soluble aAPCs that facilitate systemic administration and can expand (adoptively transferred) tumor-specific T cells directly in vivo, thereby omitting the need for ex vivo T cell activation and production.

We previously demonstrated that nanosized immunofilaments (IFs) effectively stimulate polyclonal T cell responses ex vivo. These soluble IFs are based on filamentous synthetic polyisocyanopeptides (PICs) which are rather long (∼400 nm) but very thin (∼1–2 nm) polymers and are semiflexible.^14,15^ Owing to their length, IFs can be functionalized with multiple biomolecules such as antibodies and cytokines through azides that are incorporated into the polymer side chains. Immunofilaments decorated with T cell-stimulating antibodies αCD3/αCD28 or αCD3 combined with immobilized cytokines were found to induce strong T cell activation and expansion ex vivo.^16,17^ Copresentation of αCD3 and αCD28 on the same polymer furthermore outperformed single antibodies presented on separate polymers, suggesting that combined multivalent presentation of T cell-stimulating signals is critical. In addition, we observed that these semiflexible IFs induced T cell responses were superior compared to those evoked by more rigid substrates.^16,18^ This underlines the importance of molecular flexibility of nanosized systems to facilitate the presentation of biomolecular signals to T cells in a multivalent fashion to induce potent T cell responses.^16,18^

So far, these IFs have only been applied for polyclonal T cell stimulation. Here, we focused specifically on stimulating T cells in an antigen-specific or CAR-specific manner with IFs, as this provides an opportunity for in vivo T cell activation. We demonstrate that nanosized IFs functionalized with pMHC and costimulatory molecules (αCD28 or IL-2) (Figure 1) induce strong antigen-specific T cell activation, both ex vivo and in vivo. Furthermore, IFs accumulated in lymphoid organs upon intravenous injection and displayed strong antitumor efficacy in vivo, as they inhibited metastases formation and reduced primary tumor growth in combination with immune checkpoint blockade. We conclude that IFs constitute a modular platform that can be applied as a powerful nanosized tool to trigger robust anticancer immune responses in vivo.

**Figure 1:**
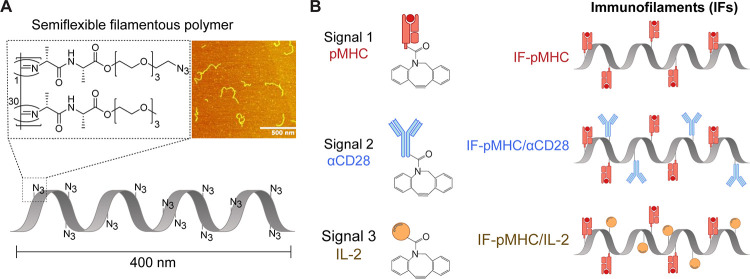
Schematic overview of the preparation of nanosized immunofilaments (IFs). (A) Chemical structure of azide-functionalized polyisocyanopeptides (PICs) (left) and atomic force microscopy image of PIC (right) with an average length of ∼400 nm and thickness of ∼2 nm. (B) Different types of IFs are prepared by conjugating dibenzocyclooctyne-functionalized biomolecules (pMHC, αCD28, IL-2) to azide-functionalized PIC.

## Results and Discussion

### Design of PIC-Based IFs

Immunofilaments for antigen-specific T cell stimulation were prepared by conjugating T cell-activating biomolecules to nanosized PIC polymers. First, azide-functionalized PICs were synthetized by copolymerization of azide-terminated isocyanopeptide monomers and nonfunctional methoxy-terminated monomers (1/30 ratio) as described previously.^17,19,20^ We obtained semiflexible PIC polymers that were ∼400 nm in length and 2 nm in width as measured by atomic force microscopy (Figure 1, inset) with a previously established statistical average of one azide group every 3.5 nm.^16,18,21^ Half of the azides were converted into biotin to enable affinity-based purification of the PIC polymers.^22^ The remaining azides were used to attach dibenzocyclooctyne (DBCO)-modified T cell-activating proteins via strain-promoted azide-alkyne cycloaddition.^23^ Using this strategy, we prepared IFs with pMHC alone or combined with agonistic anti-CD28 or recombinant IL-2 (Figure 1). The complete characterization and specifications of these polymers are provided in Tables 1 and 2 and in Figure S1.

**Table 1:** Concentration of IFs and Protein Spacing Used for Ex Vivo Experiments

immunofilament	concentration of signal 1	protein spacing on IFs
IF-pMHC^(SIIN)^	2.5 ng/mL	54 nm
IF-pMHC^(SIIN)^/αCD28		43 nm/140 nm
IF-pMHC^(SIIN)^/IL-2		43 nm/72 nm
IF-pMHC^(SIIN)^/αCD28/IL-2		49 nm/158 nm/79 nm
IF-pMHC^(SIIT)^	25 ng/mL (RNAseq: 5 ng/mL)	177 nm
IF-pMHC^(SIIT)^/αCD28		137 nm/87 nm
IF-pMHC^(SIIT)^/IL-2		137 nm/127 nm (RNAseq: 90 nm/64 nm)
IF-A2^(NYESO)^	500 ng/mL (1G4 T cells)	139 or 57 nm
IF-A2^(NYESO)/^IL-2	750 ng/mL (NY-ESO-1 TCR-transfected human T cells)	124 nm/80 or 37 nm/29 nm
IF-ProL	2 μg/mL	39 nm
IF-ProL/αCD28		20 nm/49 nm
IF-ProL/αCD28/IL-2		59 nm/142 nm

**Table 2:** Concentration of IFs and Protein Spacing Used for in Vivo Experiments

immunofilament	amount of signal 1 (ng)	protein spacing on IFs	corresponding figure
IF-pMHC^(SIIN)^ or free pMHC	290	69 nm	Figures 6A–D and 7 B,C
IF-pMHC^(SIIN)^	290	35 nm	Figures S8, S9A–E, and S10 B,C
IF-pMHC^(SIIN)^	100 – 1000	47 nm	Figure S10I,J
IF-pMHC^(SIIN)^ LD	1000	87 nm	Figure S10,E
IF-pMHC^(SIIN)^ HD	1000	26 nm	Figure S10E–G
IF-pMHC^(SIIN)^	100–1000	35 nm	Figure S10L,M
IF-pMHC^(SIIN)^	1400	50 nm	Figures 8B–D and S12
IF-pMHC^(SIIN)^	500	58 nm	Figure 8F-I
IF-A2^(NY-ESO-V)^/IL-2	1400	43 nm/58 nm	Figure S11

### Antigen-Specific Activation of Primary Mouse OT-I T Cells by IFs Ex Vivo

**Figure 2:**
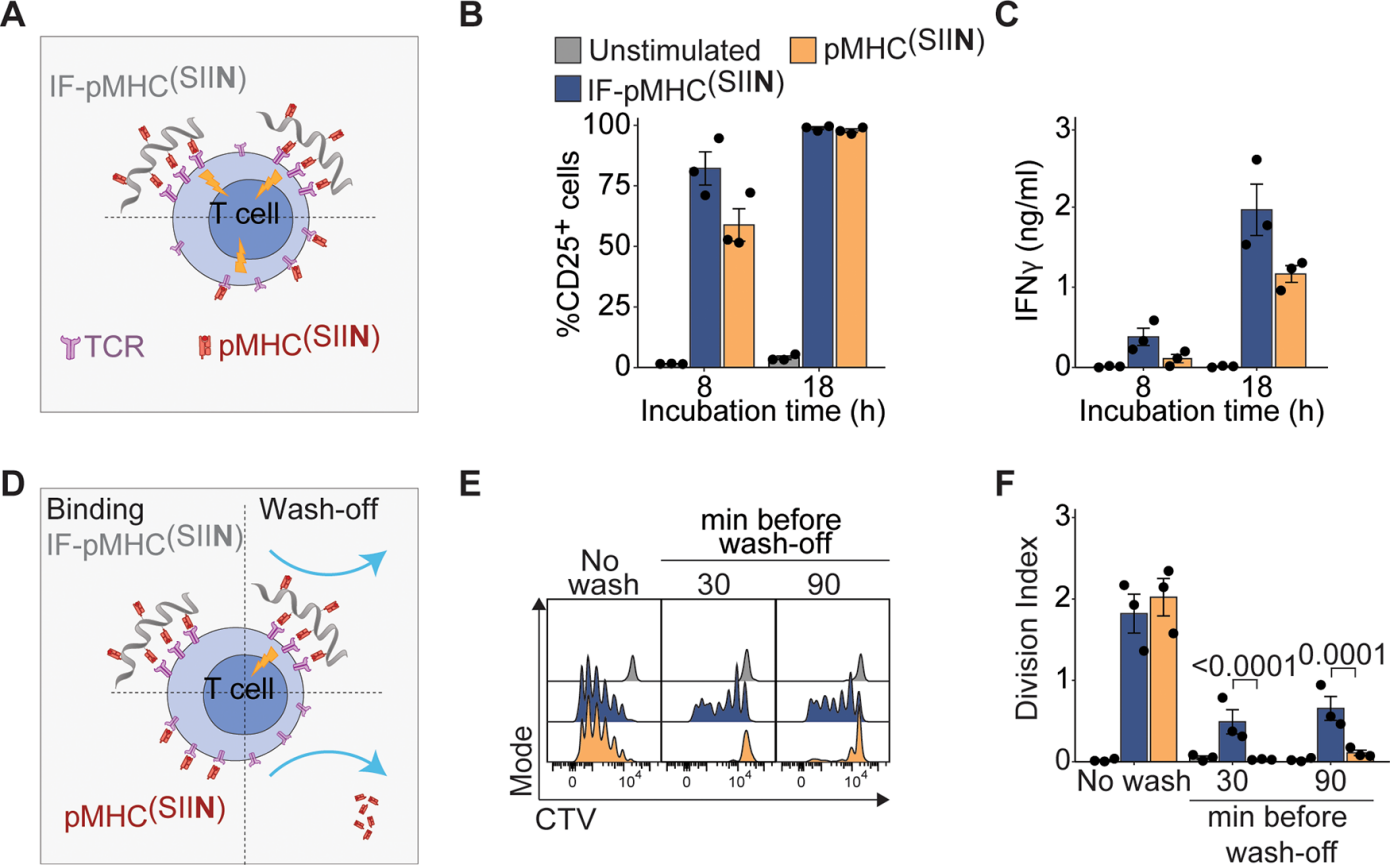
Multivalent IFs presenting pMHC activate and expand antigen-specific primary mouse T cells ex vivo. (A) Schematic overview of IF-pMHC^(SIIN)^ and free pMHC^(SIIN)^ for stimulation of murine OT-I T cells. (B) Flow cytometry quantification of the percentage of activated CD25^+^ OT-I T cells at different time points. Statistical significance was tested with two-way ANOVA on logit-transformed data. (C) The production of IFNγ by OT-I T cells at different time points was determined by ELISA. Statistical significance was tested with two-way ANOVA on log-transformed data. (D) Schematic overview of an experiment where IF-pMHC^(SIIN)^ and free pMHC^(SIIN)^ are washed off from OT-I T cells after 30 or 90 min incubation. (E,F) Representative example of the CTV dilution (E) and calculated division index of OT-I T cells (F) after three days of stimulation, either without removing the stimulation (no wash) or by washing away IF/free pMHC after 30 or 90 min, respectively. Statistical significance was determined by two-way ANOVA on log-transformed data with posthoc Sidak’s multiple comparison test. (B–C,F) *n* = 3 in three independent experiments. *p*-Values are indicated in the figure.

To validate that IFs can be used for antigen-specific stimulation of resting T cells, we used IFs presenting mouse H-2Kb-SIINFEKL (IF-pMHC^(SIIN)^) to activate freshly isolated primary OT-I CD8^+^ T cells, which express a transgenic TCR specific for the ovalbumin (OVA) epitope SIINFEKL (Figure 2A). IF-pMHC^(SIIN)^ rapidly activated OT-I cells, resulting in the expression of activation marker CD25 (Figures 2B and S2A) and production of IL-2 (Figure S2B) and interferon gamma (IFNγ) (Figure 2C) trending toward higher levels when compared with T cells that were exposed to free pMHC^(SIIN)^. Importantly, T cells stimulated with IF- pMHC^(SIIN)^ for 18 h were equally viable as T cells exposed to free pMHC^(SIIN)^ (Figure S2C).

IF-pMHC^(SIIN)^ also induced strong T cell proliferation at levels similar to T cells stimulated with free pMHC^(SIIN)^ (Figures S2D,E and S3A,C) and acquired the ability to lyse OVA-expressing B16 melanoma cells with high efficiency (Figure S2F). We furthermore observed that IF-pMHC^(SIIN)^ induced granzyme B on OT-I T cells to a significantly higher level than pMHC^(SIIN)^ alone and to similar levels as T cells stimulated in a polyclonal manner with αCD3/αCD28-presenting Dynabeads (Figure S3D–F). This clearly shows that IFs are able to induce a cytotoxic phenotype in these T cells. Characterization of their memory phenotype showed that IF-pMHC^(SIIN)^ differentiates OT-I T cells into effector memory and central memory T cells (Figure S3G). All in all, these data demonstrate the potential of nanosized IFs to expand functional T cells.

The importance of multivalency provided by the IF-pMHC^(SIIN)^ to enable stable interactions with the T cells became clear when OT-I T cells were washed after incubation with IF-pMHC^(SIIN)^ or free pMHC^(SIIN)^ (Figure 2D). Here, we found that only IF-pMHC^(SIIN)^ was able to induce IFNγ production (Figure S2G) and substantial T cell proliferation (Figure 2E,F), demonstrating that robust multivalent interactions with the IFs are essential to initiate T cell activation. We hypothesize that these multivalent interactions will be even more relevant to enable prolonged T cell stimulation in vivo following intravenous (iv) injection, as this may prevent IFs from washing off from the T cell surface by shear forces in the bloodstream.

Finally, as a result of the high affinity of OT-I TCR for the SIINFEKL ligand, we observed that solely presenting pMHC^(SIIN)^ on the IFs appeared sufficient to stimulate OT-I T cells and that copresentation of αCD28, recombinant IL-2, or both did not further boost T cell activation (Figure S2H).

### Activation and Transcriptional Characterization of Mouse T Cells Stimulated Ex Vivo by IFs Presenting Lower Affinity pMHC

**Figure 3:**
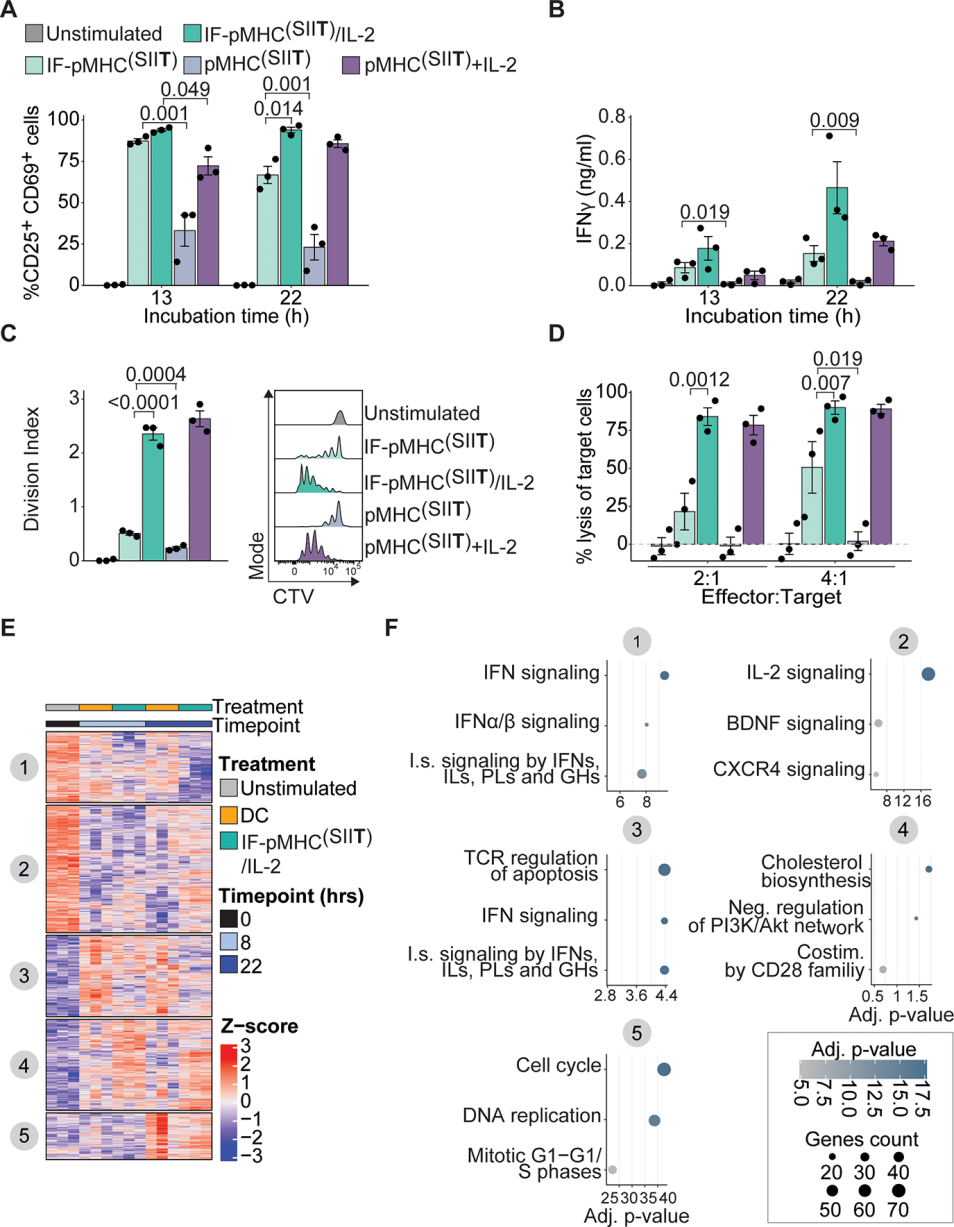
Activation and transcriptional characterization of mouse T cells stimulated by IFs presenting lower affinity pMHC. (A) Flow cytometry quantification of the percentage of activated CD25^+^CD69^+^ OT-I T cells at different time points. Statistical significance was determined with two-way ANOVA on logit-transformed data with posthoc Tukey’s multiple comparison test. (B) Production of IFNγ by OT-I T cells at different time points by ELISA. Statistical significance was determined with two-way ANOVA on log-transformed data with posthoc Tukey’s multiple comparison test. (C) OT-I T cells were stimulated for three days, after which the division index based on CTV dilution (right) was quantified as a measure of proliferation. Statistical significance was determined with one-way ANOVA on log-transformed data with posthoc Tukey’s multiple comparison test. (D) Flow cytometry quantification of the percentage of lysed B16-OVA melanoma target cells 24 h after coincubation with OT-I T cells prestimulated for 20 h. Statistical significance was determined with two-way ANOVA on logit-transformed data with posthoc Tukey’s multiple comparison test. (A–D) *n* = 3 in three independent experiments. p-Values are indicated in the figure. (E,F) Antigen-specific OT-1 I T cells were either unstimulated (0 h), or stimulated for 8 or 22 h with SIITFEKL-loaded DCs or IF-pMHC^(SIIT)^/IL-2 in triplicate in one experiment. (E) Heatmap depicting z-scores of differential genes (Benjamini-Hochberg adjusted p-value < 0.05 and fold change > 2-fold) compared to unstimulated cells (0 h). Gene clusters were obtained using k-means clustering. The columns are ordered according to treatment time point and mouse (three biological replicates per condition). (F) Gene ontology analysis of the five gene clusters, as depicted in (E), for which the three most enriched terms are visualized. X-axis and colors depict −log10 of the Benjamini-Hochberg-adjusted *p*-values for over-representation of the most enriched gene ontology terms in each cluster compared to expected. Higher values indicate stronger enrichment for the specific terms. Dot size indicates how many genes are present in the enriched gene sets. IFN: Interferon; I.s.: immune system; IL: interleukins; PL: prolactin; GH: growth hormones; BDNF: brain-derived neurotrophic factor; CXCR4: C-X-C Motif Chemokine Receptor 4; Neg. regulation: negative regulation; Costim.: costimulation; Adj.: adjusted.

We then continued to study the performance of IFs in a lower antigen affinity model, which more closely resembles antitumor T cell responses in cancer patients. To this end, we immobilized H-2Kb with the SIITFEKL peptide (pMHC^(SIIT)^), the T4 ligand for the OT-I TCR which has an approximate 10-fold lower affinity than the parental SIINFEKL ligand.^24,25^ When comparing IF-pMHC^(SIIT)^ with IFs that copresented recombinant IL-2 on the same IFs (IF-pMHC^(SIIT)^/IL-2), we observed that both IF-pMHC^(SIIT)^ and IF-pMHC^(SIIT)^/IL-2 activated OT-I T cells, as indicated by coexpression of CD69 and CD25 (Figure 3A) and induction of IFNγ production (Figure 3B), while significantly outperforming their free counterparts in this lower affinity system. Interestingly, three days after stimulation, the copresentation of IL-2 on the IFs proved to be highly favorable, as OT-I T cells stimulated with IF-pMHC^(SIIT)^/IL-2 were not only more viable (Figure S4A), but also proliferated significantly more compared to T cells stimulated with IF-pMHC^(SIIT)^ alone (an average of 2.35 cycles for IF-pMHC^(SIIT)^/IL-2 versus 0.51 cycles for IF-pMHC^(SIIT)^ in three days, Figure 3C). This beneficial effect on T cell proliferation was not observed for IF-pMHC^(SIIT)^ filaments copresenting αCD28 (Figure S4B). Moreover, IF-pMHC^(SIIT)^/IL-2 also outperformed IF-pMHC^(SIIT)^ from a functional perspective, as IF-pMHC^(SIIT)^/IL-2 clearly induced OT-I T cells with a higher target cell killing potential (Figure 3D). We conclude that copresentation of IL-2 and pMHC^(SIIT)^ immobilized on IFs enhances the proliferation and functionality of T cells expressing a lower affinity TCR. Presenting IL-2 in close proximity to a TCR trigger on IFs could furthermore be beneficial to direct IL-2 specifically to T cells and prevent off-target binding and toxicity in vivo.^17^

Next, we studied the extent to which IFs resemble natural DCs in their stimulation of antigen-specific T cells. To this end, we molecularly characterized the transcriptional programs underlying T cell activation following antigen-specific OT-I T cell stimulation with IF-pMHC^(SIIT)^/IL-2, or with primary murine DCs pulsed with the OVA protein. Both IFs and DCs induced similar expression of early activation markers CD69 and CD25 in OT-I T cells (Figure S5A-B). We then performed bulk RNA-sequencing of unstimulated OT-I T cells and of OT-I T cells after 8 and 22 h of stimulation. After filtering of lowly expressed genes, we reproducibly quantified approximately 11,000 genes in all samples. To assess how exposure of T cells to either IFs or DCs impacted their respective transcriptomes, we identified differentially expressed genes relative to the untreated control at 0 h. Hierarchical clustering of these ∼1600 genes revealed five major clusters (Figure 3E). For each cluster, we performed gene ontology analyses to classify the biological processes (Figure 3F). Both DCs and IFs stimulation activated cell cycle and cell division gene expression programs after 22 h, highlighting that similar pathways were induced and validating the activating potential of the IFs (Figure 3E,F, cluster e). Although in general very similar pathways were induced by DCs and IF, on a more detailed level we observed that T cells stimulated with IF-pMHC^(SIIT)^/IL-2 specifically upregulated genes related to IL-2 signaling at 22 h (Figure 3E,F, clusters b and d). We furthermore found that IFs stimulation evoked lower interferon signaling compared to T cells that were activated by DCs (Figure 3E,F, clusters a and c). One-on-one comparison of gene expression at 8 and 22 h after stimulation versus unstimulated OT-I T cells demonstrated that DC and IF-pMHC^(SIIT)^/IL-2 stimulation induced a similar number of upregulated and downregulated genes (Figure S5C,D). Together, these data indicate that both IF-pMHC^(SIIT)^/IL-2 and DC stimulation of T cells activate similar transcriptional profiles associated with proliferation, whereas T cells respond differently with respect to their IL-2 and IFN signaling.

### Immunofilaments Effectively Stimulate NY-ESO-1-Specific CD8^+^ T Cells and Human CD19 CAR T Cells Ex Vivo

**Figure 4:**
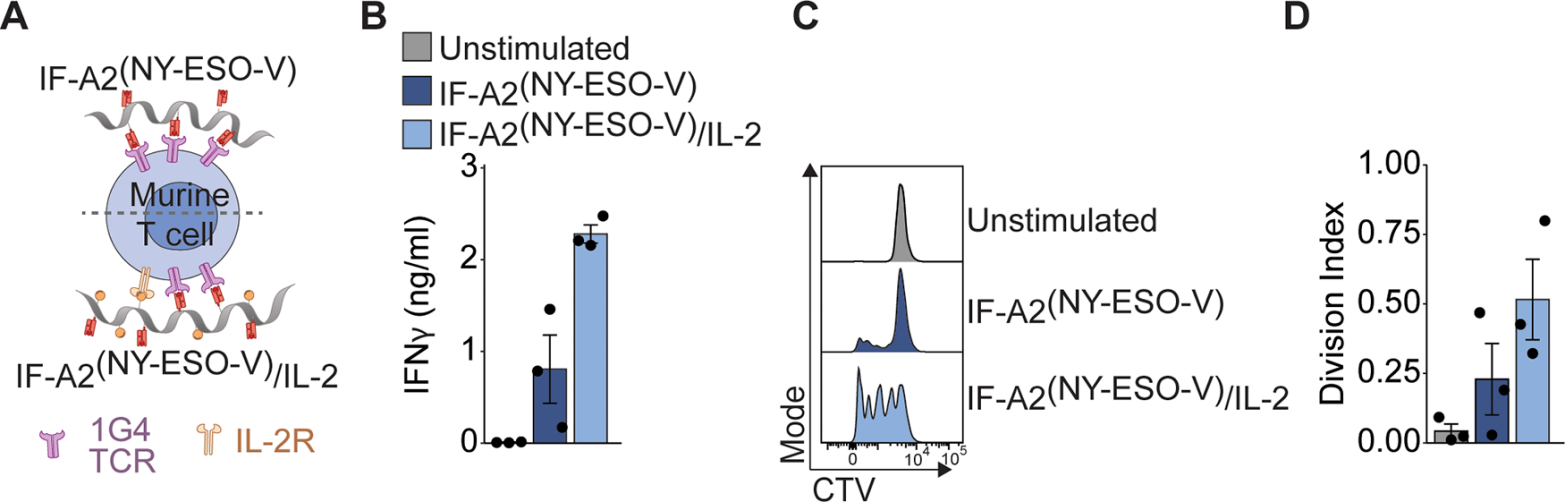
Immunofilaments effectively stimulate NY-ESO-1-specific murine CD8^+^ T cells. (A) Schematic overview of IF-A2^(NY-ESO-V)^ and IF-A2-^(NY-ESO-V)^/IL-2 for stimulation of murine 1G4 T cells. (B) IFNγ production of 1G4 T cells after 24 h of stimulation with IF-A2^(NY-ESO-V)^ and IF-A2-^(NY-ESO-V)^/IL-2. Statistical significance was tested with an unpaired *t* test on log-transformed data. (C,D) Representative example of the CTV dilution (C) and calculated division index of 1G4 T cells after three days of stimulation. Statistical significance was tested with an unpaired *t* test on log-transformed data. *n* = 3 in three independent experiments.

Next, we evaluated the versatility of the IF platform by testing their ability to activate and expand other types of antigen-specific T cells beyond the murine OT-I T cell system including a humanized transgenic T cell model and a human CAR T cell system. We are particularly interested in expanding T cells in an antigen-specific manner, as this provides the opportunity for direct in vivo T cell activation. Therefore, we focused on designs that solely supported the expansion of antigen-specific T cells. We conjugated recombinant IL-2 onto IFs together with a human HLA-A2.1-NY-ESO-1_157–165_ (SLLMWITQV). We used these IFs (IF-A2^(NY-ESO-V)^ and IF-A2^(NY-ESO-V)^/IL-2) to stimulate transgenic murine CD8^+^ T cells that express the cognate 1G4 TCR specific for the human NY-ESO-1_157–165_ (Figure 4A).^26^ The 1G4 T cells could be activated by IFs presenting A2^(NY-ESO-V)^ alone, but they displayed a profoundly enhanced production of IFNγ (Figure 4B) and proliferation (Figure 4C,D) when IL-2 was copresented (IF-A2^(NY-ESO-V)^/IL-2). These data confirm our previous observations indicating that immobilized IL-2 can enhance CD8^+^ T cell responses when it is copresented with pMHC on IF.

Next, we stimulated human CD8^+^ T cells transfected with a transgenic TCR against NY-ESO-1 (Figure S6A), and we observed an increase in their expression of CD25 (Figure S6B). We furthermore found that only TCR-transfected but not mock-transfected human T cells produced IFNγ and proliferated in response to IFs (Figure S6C–F), demonstrating the antigen-specificity of the IF.

**Figure 5:**
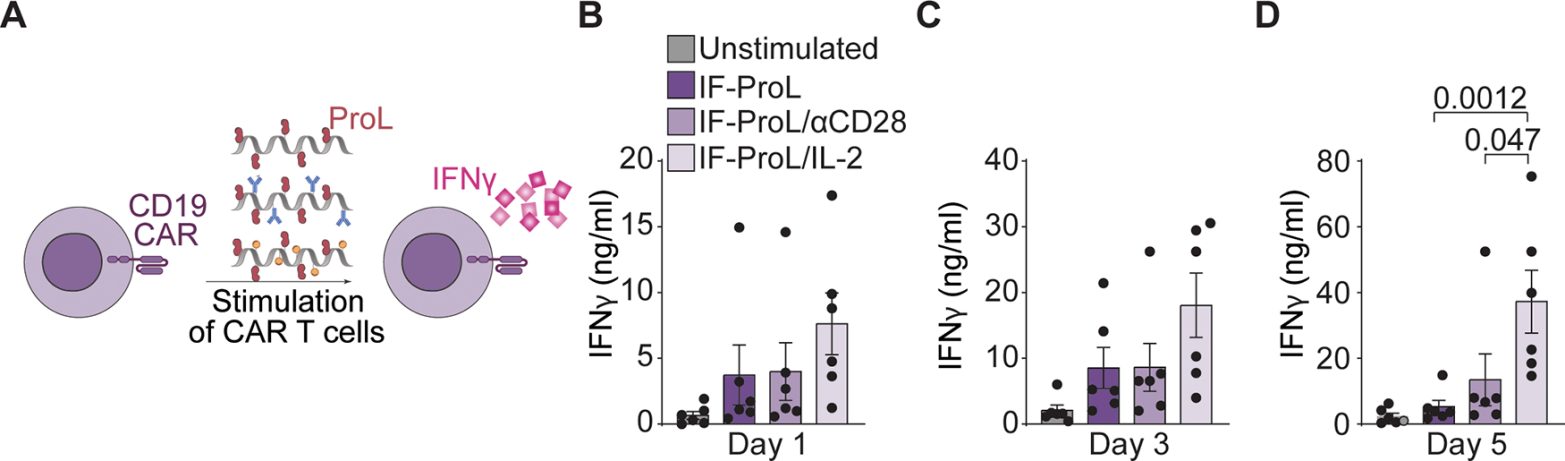
Immunofilaments effectively stimulate human CD19 CAR T cells ex vivo. (A) Schematic overview of IF-ProL, IF-ProL/αCD28 and IF-ProL/IL-2 for the stimulation of human T cells lentivirally transduced with a CD19 CAR construct. (B–D) IFNγ production after stimulation with IFs for 1 day (B), 3 days (C), and 5 days (D). Statistical significance was determined by two-way ANOVA on log-transformed data with posthoc Tukey’s multiple comparison test. *n* = 6 in six independent experiments. *p*-values are indicated in the figure.

Artificial APCs are used frequently for the bulk ex vivo expansion of T cells genetically engineered to express CARs, after which these potentiated T cells are infused back into patients for immunotherapeutic purposes. CAR T cell expansion usually relies on αCD3/αCD28-coated Dynabeads for T cell stimulation. Although this polyclonal stimulation can efficiently expand CAR T cells, bystander non-CAR T cells that can constitute a significant fraction are expanded at equal rates. This does not only reduces therapy efficacy but could also pose threats with respect to unwanted side effects after infusion.^27^ We therefore probed the potential of the IFs to selectively expand CD19-directed CAR T cells by activating them through the engagement of the CARs. We produced IFs that present Protein L (ProL), which binds to the variable kappa light chains of immunoglobulins and thereby specifically cross-links any CAR on the T cell surface to induce T cell activation.^28,29^ In addition, we prepared IFs that copresented ProL and anti-human CD28 or recombinant IL-2 (Figure 5A). A mixture of CD4^+^ and CD8^+^ T cells was lentivirally transduced with a second generation CD19 CAR construct.^30^ After expansion and resting, the CD19 CAR T cells were restimulated with IF. We observed increased IFNγ production by CAR T cells stimulated with IF-ProL/IL-2 compared to those stimulated with IF-ProL or IF-ProL/αCD28 (Figure 5B–D), resulting in 6- and 2-fold enhanced IFNγ levels on day 5, respectively. Whereas αCD28 did not seem to have any beneficial effect on CAR T cell activation, the copresentation of ProL with IL-2 synergized to boost CAR T cell stimulation (Figure S7), again demonstrating that coimmobilizing and TCR-triggering molecule and IL-2 enhances T cell proliferation.

Together, these data exemplify the versatility of IFs in their ability to effectively activate different types of antigen-specific T cells with different affinities and genetically engineered T cells. For CAR T cells, ProL-presenting IF allow for specific expansion of CAR-bearing T cells only, thereby limiting the number of nonspecific T cells in the infusion product. The IFs could furthermore be applied for in vivo rather than ex vivo stimulation of CARs, thereby preventing the differentiation and loss of anticancer activity that has been described when CARs are expanded ex vivo.^31^ As such, IFs constitute a modular platform for the antigen-specific/CAR-specific expansion of T cells, for which the presented biomolecules can be easily adjusted to the requirements of the system.

### In Vivo Biodistribution of IFs Following Intravenous Administration

**Figure 6:**
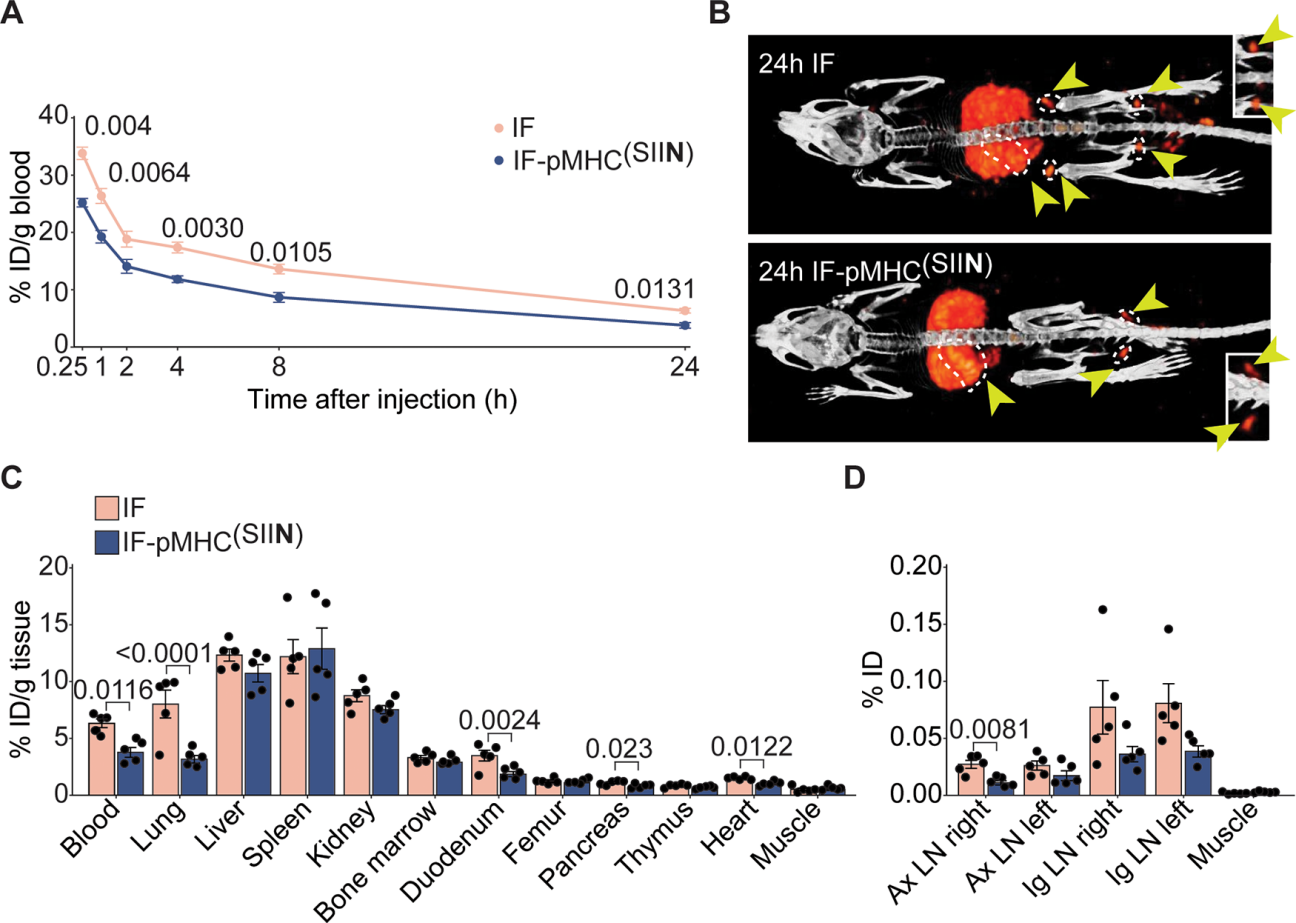
In vivo biodistribution of IFs after intravenous injection. (A) Kinetics of ^111^In-labeled IFs and IF-pMHC^(SIIN)^ in the blood after iv injection in WT C57BL/6 mice, expressed as % of injected dose (ID) per gram of blood. *n* = 6 for *t* = 0.25, 1, 2, 4 h and *n* = 5 for *t* = 8 and 24 h in one independent experiment. Statistical significance was determined with mixed effect analysis and posthoc Sidak’s multiple comparison test; *p*-values are indicated in the figure. (B) 2D maximum intensity projection SPECT/CT images 24 h after iv injection of ^111^In-labeled IFs and IF-pMHC^(SIIN)^. Yellow arrows indicate secondary lymphoid organs (dotted white line indicates spleen and lymph nodes). Inset: Magnification of the popliteal lymph nodes. (C) Quantitative ex vivo analysis of the biodistribution of IFs 24 h after iv injection across different organs expressed as % of ID per gram of tissue. Statistical significance was determined by one-way ANOVA on log-transformed data with posthoc Sidak’s multiple comparison test. *n* = 5 in one independent experiment. p-Values are indicated in the figure. (D) Quantitative ex vivo analysis of the % of the ID of IFs 24 h after iv injection in the axillary lymph nodes (Ax LN) and inguinal lymph nodes (Ig LN), compared to a piece of quadriceps muscle with the same weight. Statistical significance was determined by one-way ANOVA on log-transformed data with posthoc Sidak’s multiple comparison test. *n* = 5 in one experiment. *p*-Values are indicated in the figure.

Before applying IFs therapeutically for in vivo expansion of T cells, we assessed their biodistribution after iv administration to analyze whether they could reach lymphoid organs. We labeled nonfunctionalized IFs and IF-pMHC^(SIIN)^ with ^111^In and injected them iv into mice. Mice did not show any discomfort following injection. Both nonfunctionalized IFs and IF-pMHC^(SIIN)^ were detectable in the blood for up to 24 h after administration though IF-pMHC^(SIIN)^ was cleared from the systemic circulation slightly faster (6.3% ID/g for nonfunctionalized IFs versus 3.8% ID/g for IF-pMHC^(SIIN)^, Figure 6A). Both IFs displayed similar biodistribution patterns across the organs after 24 h, with substantial accumulation in spleen, liver, lungs, and kidneys (Figure 6B,C). In addition to the significant accumulation of nonfunctionalized IFs and IF-pMHC^(SIIN)^ in the spleen, we also observed notable amounts of IFs in other secondary lymphoid organs in which T cells predominantly reside, including the popliteal (Figure 6B), the axillary, and inguinal lymph nodes (Figure 6D). This facilitates the interaction of IFs with antigen-specific T cells, which is essential to trigger T cell responses in vivo. When we studied in more detail which cells within the spleen take up the IF, we observed that both IF and IF-pMHC^(SIIN)^ were present in CD11c^+^ and CD11b^+^ cells, but the dose given was below the detection limit to be observed in CD3^+^ cells after 24 and 72 h (Figure S8).

To investigate whether the biodistribution of IF-pMHC^(SIIN)^ would be affected by the presence of antigen-specific T cells in vivo, we administered ^111^In IF-pMHC^(SIIN)^ iv to mice that received adoptively transferred WT T cells or OT-I T cells. The biodistribution of IF-pMHC^(SIIN)^ was not altered by the presence of antigen-specific OT-I T cells, apart from a slightly higher accumulation of IF-pMHC^(SIIN)^ in the spleen, which may suggest that IF-pMHC^(SIIN)^ interacts with and is retained in the spleen by OT-I T cells (Figure S9). Taken together, these data demonstrate that nonfunctionalized IFs and IF-pMHC^(SIIN)^ have similar biodistribution patterns, are available for > 24 h in the blood and can reach lymphoid organs.

### Immunofilaments Induce Antigen-Specific T Cell Proliferation In Vivo

**Figure 7:**
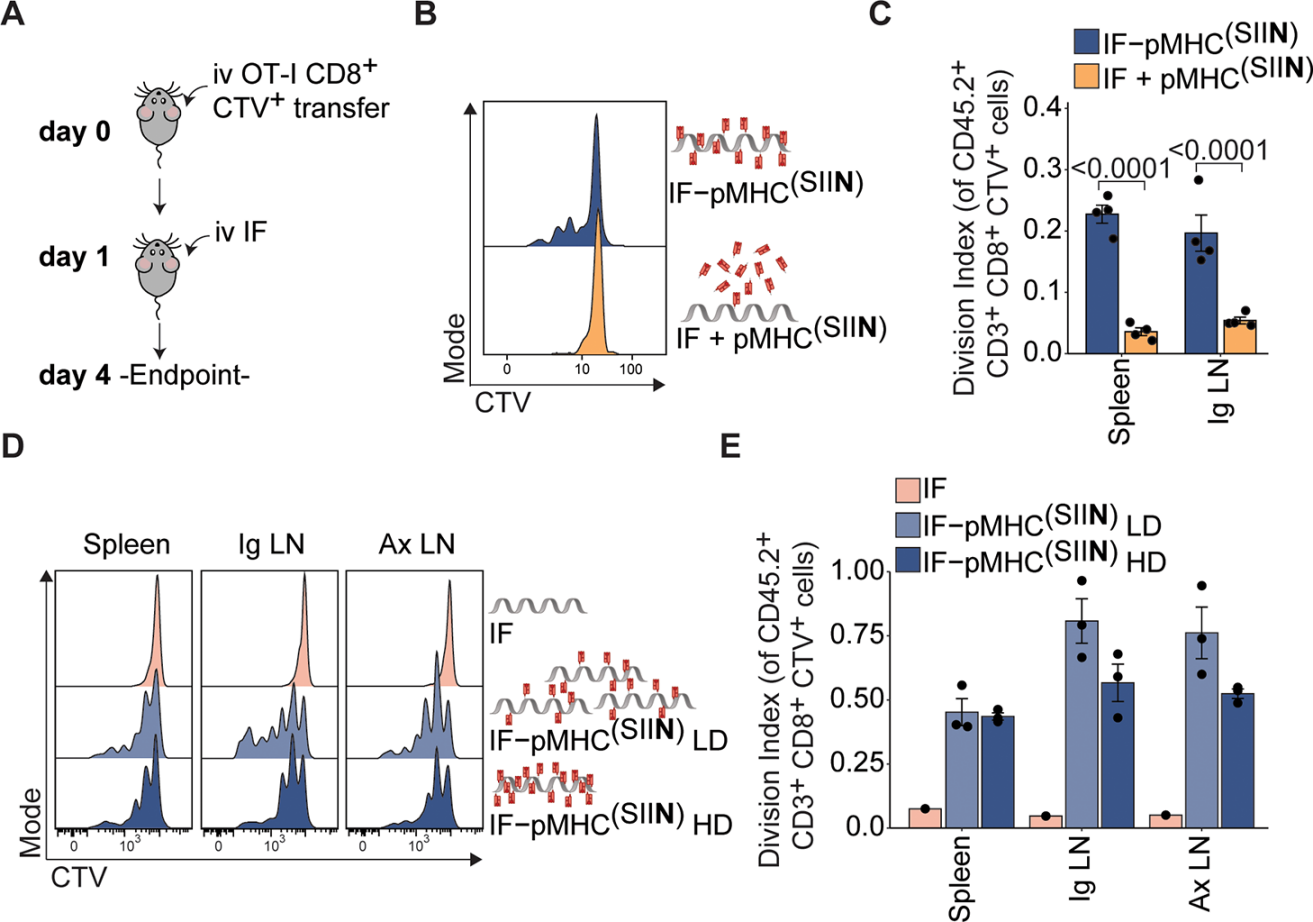
Immunofilaments expand antigen-specific T cells in vivo. (A) Schematic overview of experimental setup. (B,C) Ex vivo flow cytometric analysis of CTV dilution (B) and quantification (C) of the division index of CD45.2^+^CD3^+^CD8^+^ OT-I T cell in the spleen 3 days after iv administration of 0.29 μg free pMHC^(SIIN)^ + IFs or IF-pMHC^(SIIN)^ in CD45.1^+^ WT C57BL/6 adoptively transferred with 5 × 10^5^ OT-I T cells. Statistical significance was determined on log-transformed data with one-way ANOVA and posthoc Sidaks’s multiple comparison test. *n* = 4 in one independent experiment. (D,E) Ex vivo flow cytometric analysis of CTV dilution (D) and quantification (E) of the division index of OT-I T cells in the spleen and inguinal (Ig) and axillary (Ax) lymph nodes 4 days after iv administration of IFs alone or IF-pMHC^(SIIN)^ with a low density (LD): ∼5 pMHC per IF) or high density (HD): ∼16 pMHC per IF). The total amount of pMHC^(SIIN)^ (1 μg) that was administered was kept constant. Statistical significance was tested with two-way ANOVA on log-transformed data with Tukey’s multiple comparison test. *n* = 1–3 in one independent experiment.

The observation that IFs readily reach lymphoid organs upon administration in vivo (Figure 6) provided us with the opportunity to exploit IFs for direct T cell expansion in vivo. To this end, we adoptively transferred unstimulated OT-I T cells into mice and 24 h later injected IF-pMHC^(SIIN)^ or nonfunctionalized IFs mixed with free pMHC^(SIIN)^ (Figure 7A). The IF-pMHC^(SIIN)^ induced proliferation of OT-I T cells in vivo to a much higher extent than when these two components were given separately (Figure 7B,C). These findings confirm the importance of providing T cell-activating biomolecules on a polymer backbone to facilitate docking to T cells and support downstream signaling by multivalency, probably combined with the effect of a more favorable half-life and biodistribution for the IF-pMHC^(SIIN)^. When IF-pMHC^(SIIN)^ was administrated to mice that received adoptive transfer of wild-type CD8^+^ T cells instead of antigen-specific OT-I T cells, we did not observe any proliferation (Figure S10A–C). These findings clearly demonstrate that IF-pMHC^(SIIN)^ triggers T cell activation in an antigen-specific manner in vivo. Furthermore, IF-A2^(NY-ESO-V)^/IL-2 was also able to trigger proliferation of antigen-specific 1G4 T cells that were adoptively transferred into recipient mice (Figure S11), showing that this observation also holds true for other antigens and other T cells expressing different TCRs.

To investigate how IFs design affects T cell proliferation, we next compared proliferation of adoptively transferred OT-I T cells after injection of IF-pMHC^(SIIN)^ decorated with different numbers of pMHC^(SIIN)^. Keeping the amount of injected pMHC^(SIIN)^ constant, we varied the amount and spacing of pMHC^(SIIN)^ per polymer (low density (LD): ∼5 pMHC per IF, high density (HD): ∼16 pMHC per IF) and thus varied the total amount of IFs that we administered to the animals. As we observed that the nonfunctionalized IFs backbone itself does not induce OT-I T cell proliferation (Figure 7D,E), this allowed us to study the impact of the pMHC^(SIIN)^ density on IFs in vivo. We observed a slightly higher proliferation of OT-I T cells upon administration of LD IF-pMHC^(SIIN)^ compared to HD IF-pMHC^(SIIN)^ in the lymph nodes but not in the spleen, which indicated that a lower density of pMHC^(SIIN)^ is sufficient for T cell activation (Figure 7D,E). We hypothesize that a low density of pMHC^(SIIN)^ already being effective is the direct consequence of the administration of a higher number of IFs molecules in total, thereby increasing the chances of IF-pMHC^(SIIN)^ to meet and interact with antigen-specific T cells in vivo. Next, we studied the importance of the time interval between adoptive cell transfer of OT-I T cells and injection of IF. We found that for both LD and HD IFs the resulting T cell proliferation was similar irrespective of whether IF-pMHC^(SIIN)^ were administrated 10 min, 4 h, or 24 h after adoptive cell transfer (Figure S10D–G). Finally, we studied how the number of adoptively transferred OT-I T cells and the dose of IF-pMHC^(SIIN)^ affect T cell proliferation in vivo after iv administration of IFs (Figure S10H). Whereas the number of transferred OT-I T cells did not affect their proliferation, we observed a clear effect of the IF-pMHC^(SIIN)^ dose by iv injection on the level of OT-1 proliferation in terms of both the percentage of proliferating cells and their division index (Figure S10I,J). Within the tested dose range, we did not observe dose-dependent proliferation when IFs were administered subcutaneously (sc) as there was high proliferation for all doses tested, suggesting that the sc delivery of IFs is highly efficient (Figure S10K–M). In conclusion, IFs are able to stimulate antigen-specific T cells in vivo through iv and sc administration, which leads to T cell expansion.

### Immunofilaments Inhibit Primary Tumor Growth and Reduce Metastatic Spread In Vivo

**Figure 8:**
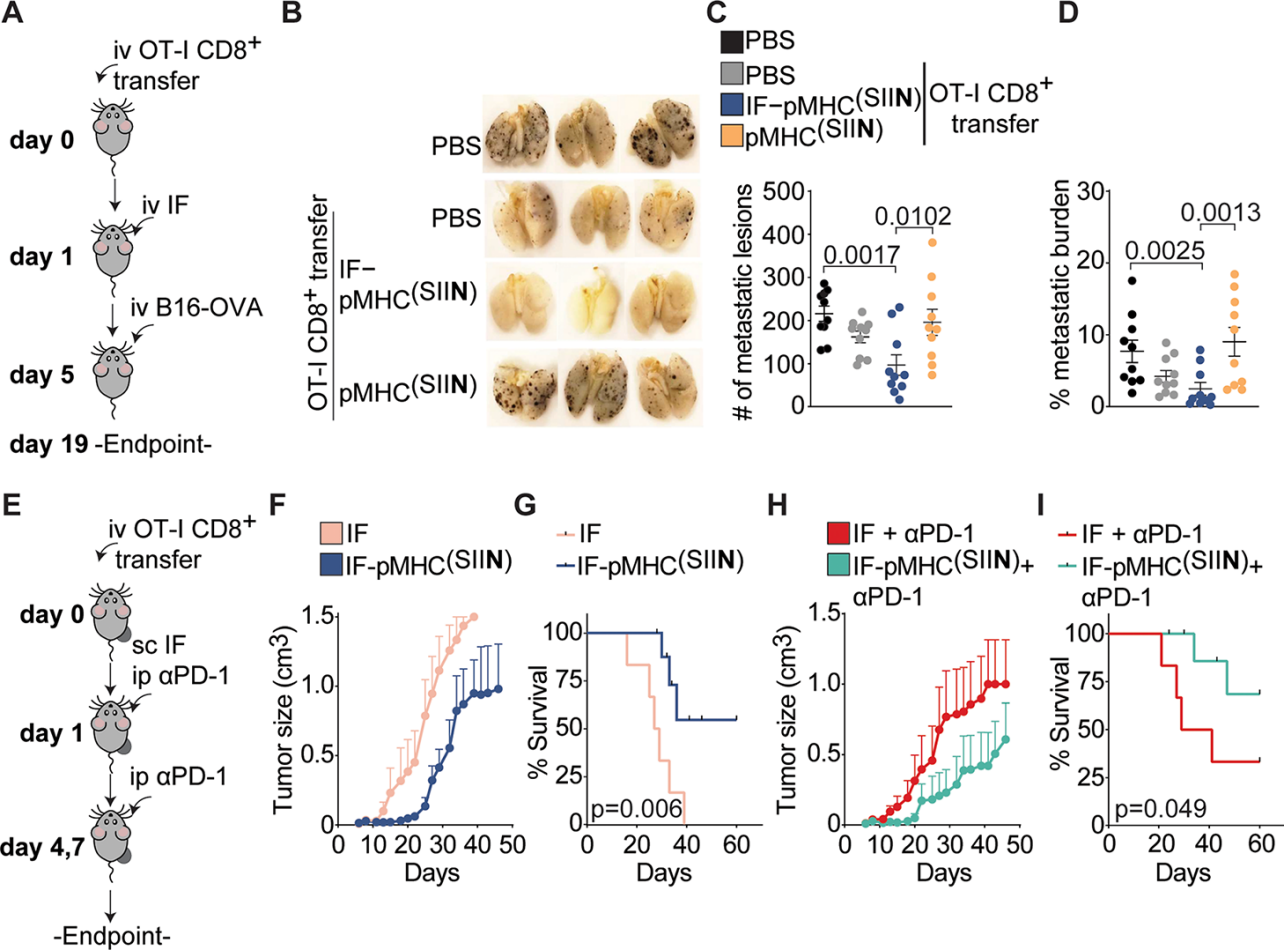
In vivo administration of IFs inhibits primary tumor growth and prevents the development of metastases. (A) Overview of experimental setup of the lung metastasis model. OT-I T cells were adoptively transferred into WT mice, followed by a single iv injection of IFs on day 1. On day 5, B16-OVA tumor cells were injected iv and on day 19 the lungs were collected and fixed overnight in Feketés solution. The number of metastatic nodules in the lungs was enumerated. (B,C) Representative overview of metastatic lesions (in brown/black) in the lungs at day 19 (B) and enumeration of the total number of metastatic nodules in the lungs (C). *n* = 10 in 2 independent experiments. Statistical significance was determined with one-way ANOVA and posthoc Dunnet’s multiple comparison test. (D) Quantification of the metastatic burden of the lungs on day 19, defined as the percentage of the lungs occupied by metastatic lesions. Statistical significance was determined by one-way ANOVA on logit-transformed data and posthoc Dunnet’s multiple comparison test. (E) Overview of the experimental setup of the sc injection of IF. OT-I T cells were adoptively transferred into B16-OVA tumor-bearing mice, followed by a single sc injection of IFs on day 1 with or without three times intraperitoneal injection of αPD-1. (F,G) Quantification of the size of B16-OVA tumors (F) and the survival (G) of mice treated with nonfunctionalized IFs or IF-pMHC^(SIIN)^. *n* = 6–9 in one independent experiment. (H,I) Quantification of the size of B16-OVA tumors (H) and the survival (I) of mice treated with IFs + αPD-1 or IF-pMHC^(SIIN)^ + αPD-1. (G, I) Statistical significance was determined with the Gehan-Breslow-Wilcoxon test. Mice that did not reach the tumor size of 1500 mm^3^ were marked as censored. *n* = 6–9 in one experiment.

Finally, we investigated the potential of IFs in vivo in the context of cancer models. We made use of a well-established aggressive melanoma model in which the effect of IFs on the establishment of pseudometastasis can be probed. As our aim is to activate T cells directly in vivo without the need for preactivation, freshly isolated OT-I T cells were adoptively transferred into mice and stimulated through iv administration of IF-pMHC^(SIIN)^ or free pMHC^(SIIN)^ (Figure 8A). On day five, B16-OVA melanoma cells were injected iv into the tail vein to induce the development of metastases in the lungs, and 19 days postinjection, lungs were isolated and pulmonary lesions enumerated (Figure 8B). Although the transfer of OT-I T cells alone modestly reduced the number of metastatic lesions in the lungs (an average of 162 metastatic nodules), iv treatment with IF-pMHC^(SIIN)^ significantly decreased the number of metastatic nodules to an average of 97 (Figure 8C) compared with PBS treatment alone. In vivo stimulation of adoptively transferred OT-I T cells with IF-pMHC^(SIIN)^ proved to be equally effective in reducing the number of metastatic lesions as the administration of ex-vivo preactivated OT-I T cells, without the need for the laborious ex-vivo stimulation of T cells (Figure S12A). The administration of free pMHC^(SIIN)^ did not lead to a reduction in the number of metastatic lesions compared to that of the controls, which is probably the result of fast clearance and poor OT-I T cell stimulation. Importantly, the body weight of the mice was not negatively impacted by treatment with IF-pMHC^(SIIN)^ during the experiment (Figure S12B), suggesting that the IF-pMHC is well-tolerated and can be safely administered in vivo. We next examined the lungs to establish the total metastatic burden, which we defined as the percentage of the lung area occupied by metastatic lesions. This analysis not only considers the number of metastatic lesions but also incorporates the size of the individual nodules, and we again found that IF-pMHC^(SIIN)^ significantly outperforms pMHC^(SIIN)^ in reducing the metastatic burden (Figure 8D).

To investigate how IFs perform in a therapeutic cancer model where an established tumor is present, we adoptively transferred OT-I T cells into mice bearing B16-OVA melanoma tumors in their flank. Next, a single dose of IF-pMHC^(SIIN)^ was administrated in sc in close proximity to the established tumors (Figure 8E). We opted for sc administration instead of iv injection as we had previously established that this results in higher levels of proliferation in adoptively transferred OT-I T cells (Figure S10H–M). Mice receiving a single dose of IF-pMHC^(SIIN)^ displayed a substantial delay in tumor growth and a significantly enhanced survival compared with mice that received IFs alone (Figures 8F,G and S13A–E). We next studied if stimulation and proliferation of antigen-specific T cells with IF-pMHC^(SIIN)^ could synergize with alleviating immune suppression through blocking of immune checkpoint PD-1 on T cells. To this end, we combined the sc administration of IF-pMHC^(SIIN)^ with intraperitoneal injections of αPD-1. The results confirmed that this combination provides a clear benefit in terms of delaying tumor growth and significantly improving survival compared to treatment with αPD-1 alone (Figure 8H,I). Histological analysis of the T cell infiltration in these tumors showed that mice that were treated with either IF-pMHC^(SIIN)^ or IF-pMHC^(SIIN)^ + αPD-1 had slightly elevated numbers of CD8^+^ T cells infiltrating their tumors (Figure S13F-J). These results clearly indicate that OT-I T cells stimulated by a single dose of IFs are highly functional and migrate to the tumor where they can combat the growth of primary B16-OVA tumors in vivo.

Altogether, our findings demonstrate that nanosized IFs constitute a powerful tool that elicits strong T cell activation ex vivo and in vivo, resulting in potent antitumor efficacy. Previously, other aAPCs have been developed that can be administered in vivo to stimulate T cells, but these aAPC platforms predominantly consist of microsized beads.^32−36^ Their large surface area and low surface curvature promotes interaction with the T cell surface, resulting in strong T cells responses that cannot be obtained with nanosized beads.^37,38^ However, their micrometer range size prohibits effective intravenous injection as they are rapidly cleared by the reticuloendothelial system, have limited access to lymphoid organs and can pose significant safety concerns.^37,38^ Instead, IFs provide a promising alternative, as they are nanosized and at the same time give rise to strong T cell activation and initiate proliferation programs. We hypothesize that this is due to the semiflexible nature of the filaments, which supports receptor clustering on the cell surface to drive signaling when biomolecules are presented multivalently to T cells. Consequently, our nanosized IFs do not suffer from the challenges associated with iv delivery of microsized beads but instead remain circulating in the blood for at least 24 h following iv administration and can effectively reach lymphoid organs. These IFs effectively expand antigen-specific T cells in vivo that acquire tumor-killing capacities and are able to migrate, thereby delaying tumor growth and extending survival. We furthermore demonstrate IFs can be applied for both iv and sc injection, putting forward IFs as a modular and broadly applicable platform adding to the arsenal of immunotherapies to fight cancer. The IFs complement other approaches that aim to activate (CAR) T cells in vivo, such as stimulation by CD19-loaded extracellular vesicles^39^ or vaccination with amphiphilic CAR T cell ligands that decorate the surface of APCs in the LN.^40^

## Conclusions

We have developed a nanosized IF platform that is able to present pMHC, costimulatory molecules, and cytokines in a multivalent manner, which effectively stimulate antigen-specific T cells. Our findings demonstrate that nanosized IFs elicit strong T cell activation ex vivo and initiate transcriptional programs largely similar to those evoked by natural DCs. We furthermore observed that coimmobilization of IL-2 and pMHC on IFs evokes particularly potent T cell responses for lower affinity T cell antigens. These IFs constitute a versatile platform that can be applied for the ex vivo expansion of antigen-specific T cells or CAR T cells. The most important benefit of these nanosized IFs is that owing to their small size, they can readily be applied to activate and expand T cells in vivo through iv and sc injection. After iv injection, IFs can reach lymphoid organs and evoke strong T cell responses in vivo. As a result, even a single dose of IFs has a strong antitumor effect, as it inhibits the formation of metastases in a melanoma model and reduces primary tumor growth in synergy with immune checkpoint blockade. We conclude that these nanosized IFs are a powerful and versatile type of aAPC. They constitute a major step forward in the treatment of cancer as in vivo administration of IFs may not only specifically activate and expand existing tumor specific T cells or TIL, but may also revitalize T cells or CAR-T cells in vivo that were previously administered by ACT, thus enhancing their lifespan and therapeutic efficacy.

## Methods

### Immunofilament Synthesis and Characterization

IFs were prepared as described before.^17,19,20^ Briefly: isocyanopeptide monomers with nonfunctional methoxy and functional azide groups were polymerized in a 30:1 ratio using a nickel catalyst (1/10 000 ratio), yielding polymers with statistically one azide group every 3.5 nm.^16,18,21^ Next, 60% of the azides were reacted with DBCO-PEG4-biotin according to literature procedures.^22^ The average length of the azide/biotin polymers was determined using atomic force microscopy (AFM, nanoscope III, digital instruments) operated in tapping mode in air. The polymers were dissolved in MilliQ (10 μg/mL) and drop casted on freshly cleaved mica for 5 min, after which the sample was dried under a nitrogen flow. From the resulting images the polymer length was determined using ImageJ.^41^ The average length determined was 407 ± 207 nm, calculated from 161 values. DLS could not be performed on IFs as they are linear thread-like polymers with a semiflexible character and no adequate models for fitting of the data are available.

### MHC Production

MHC were prepared as described.^42^ The constructs for the heavy chains (HLA-A2, H-2Kb) and human beta-2-microglobulin (hβ2m) were generously provided by M. Toebes and T.N. Schumacher from the NKI. They were produced as inclusion bodies in E.coli BL21(DE3)pLysS using T7 RNA polymerase/promoter system.^43^ Isolated inclusion bodies were solubilized in a denaturing buffer (8 M urea/100 mM Tris·Cl, pH 8). Hβ2m was prefolded in dialysis against 10 mM Tris·Cl (pH 7) in PBS. To prepare the final MHC complex, hβ2m and heavy chains were dissolved to final concentrations of 6 and 3 mM, respectively, in folding buffer (100 mM Tris·Cl, pH 8; 400 mM l-arginine; 2 mM EDTA; 5% glycerol; 5 mM reduced glutathione; 0.5 mM oxidized glutathione; Protease Inhibitor Cocktail, Roche Diagnostics) with a 60 mM template peptide (NY-ESO-V: SLLMWITQV, which is a higher affinity analogue of the natural NY-ESO-1_157–165_ peptide where the terminal cysteine (C) amino acid is replaced by valine (V),^44^ SIINFEKL, SIITFEKL; GenScript). The folding reaction mixture was incubated at 10 °C for 5 days. After filtration, concentration, and buffer change to PBS, the complexes were purified via size exclusion chromatography using a HiLoad 16/600 Superdex 75pg column (Cytiva). Ready MHC was analyzed using SDS-PAGE and NanoDrop, concentrated, snap-frozen, and stored at −80 °C until further use.

### Protein Functionalization

Proteins were functionalized using previous reported protocols.^9,17^ Briefly, pMHC^(SIIN)^, pMHC^(SIIT)^, and A2^(NY-ESO-V)^ were obtained from the refolding protocol, typically 0.5–2 mg/mL in PBS (pH 7.4). Mouse αCD28 and human αCD28 were obtained from BioXcell, IL-2 was obtained from ProSpec and ProL was obtained from Acrobiosystems. Before use, mouse αCD28, human αCD28 and ProL were washed with borate buffer (pH 8.4, 50 mM) using 30 kDa spinfilters from Amicon, typically to 2–3 mg/mL. IL-2 was first reconstituted in MilliQ (10 mg/mL) and after 10–30 min further diluted with PBS (pH 7.4), typically to 1 mg/mL. To functionalize the proteins, DBCO-PEG4-NHS (Click chemistry tools, 100 mM in DMSO) and dye-NHS (10 mM in DMSO) were added to the protein stock solutions; typically 3–4 equiv of DBCO and 2.5–3 equiv of dye were used. Dyes used in this study are AlexaFluor350-NHS (Thermo Fisher Scientific), Atto488-NHS (AttoTec), AlexaFluor594-NHS (Thermo Fisher Scientific), and AZdye647-NHS (click chemistry tools). For proteins in borate buffer, reactions were run for 2–3 h at 4 °C. Proteins in PBS were left to react for 4–6 h at 4 °C. After the reaction, the proteins were purified using spin filtration against PBS with spin filters of appropriate size (Amicon). The protein conjugates were analyzed with NanoDrop (Thermo Fisher Scientific), using the following extinction coefficients: MHCs (95 000 M^–1^ cm^–1^), mouse/human αCD28 (210 000 M^–1^ cm^–1^), ProL (35 760 M^–1^ cm^–1^), IL-2 (11 900 M^–1^ cm^–1^), DBCO (12 000 M^–1^ cm^–1^), AlexaFluor350 (19 000 M^–1^ cm^–1^), Atto488 (90 000 M^–1^ cm^–1^), AlexaFluor594 (120 000 M^–1^ cm^–1^), and AZdye647 (270 000 M^–1^ cm^–1^). Data were measured at 280 nm (protein), 309 nm (DBCO) and the wavelength of the dye. The raw data was corrected for spectral overlap between the different components using correction factors as described.^17^ Typical obtained degrees of labeling (DOL) for DBCO was 0.5–3 and 0.5–2 for dyes.

### Immunofilament-Protein Conjugates

The DBCO/dye functionalized proteins were coupled to the IFs using a protocol as described before.^9,17^ Briefly, 100–200 μL of a stock solution of 1 mg/mL biotin/azide polymer was reacted with the required amount of protein in PBS (pH 7.4), typically 0.2–1 equiv of protein irt free azides was used. All reactions were carried out in nonstick Eppendorf tubes (Fisher Scientific), with a final concentration of 0.2–0.25 mg/mL polymer. Reactions were first mixed for 4–5 h at RT, followed by incubation at 4 °C overnight. Purification was performed following a literature protocol, using monoavidin resin (Thermo Fisher Scientific).^22^ Per 100 μg of polymer, 1 mL of monoavidin resin was used. After 2x washing of the resin with PBS (pH 7.4), the resin was added to the reaction mixtures and incubated for 1.5–2 h at 4 °C. Next, the resin was washed with 1x PBS-tween (0.1%) and 4–5x PBS. After washing, a solution of 2 mM biotin in PBS was added (300–400 μL) to elute the polymer bioconjugates from the monoavidin resin (1–2 h incubation at 4 °C). Concentrations of the conjugated proteins were determined using fluorescence (Tecan spark 10 M plate reader), using the soluble labeled proteins as the standard. Polymer concentration was determined using circular dichroism spectroscopy (JASCO J-810), from which a standard curve was inferred (Figure S1) to determine the loading amount of coupled proteins. With these concentrations the average spacing/density of the protein on the polymer could be calculated, using the fact that every monomer is 0.115 nanometer in size^45^ (Tables 1 and 2). We found that polymers with proteins attached can be used for at least 1 year when stored at −20 °C (Figure S2H).

### Preparation of ^111^In-Immunofilaments

To label IFs with ^111^In, first diethylenetriaminepentaacetic acid (DTPA)-PEG4-DBCO was prepared. *p*-NH_2_-Bn-DTPA (Macrocyclics) was dissolved in 100 μL of DMF (0.013 mmol, 8.6 mg), and 87 μL of a 100 mg/mL solution DBCO-PEG4-NHS in DMF was added (0.013 mmol, 8.7 mg). Next, 30 μL of triethylamine was added, and the reaction was mixed overnight at room temperature. Formation of the product was confirmed by using MALDI-ToF with α-cyano-4-hydroxycinnamic acid as the matrix. The crude reaction mixture was further purified using HPLC with a triethylammonium bicarbonate buffer/methanol solvent system (0.9 mg, 0.87 μmol, 9%). MALDI-ToF *m*/*z* calculated for C_51_H_65_N_6_O_17_ [M+H]^+^ 1033.441, found 1033.272. Next, DTPA-PEG4-DBCO was dissolved in MilliQ to a 9 mg/mL concentration and added during the coupling of proteins to the IFs when needed; typically 2 equiv of DTPA-PEG4-DBCO irt free azides was used.

Labeling of the DTPA-bearing IFs with ^111^In was performed by using the following protocol: DTPA-IFs were incubated with [^111^In]InCl_3_ in 0.5 M 2-(*N*-morpholino)ethanesulfonic acid (MES) buffer, pH 5.5, at 37 °C, under rotation (550 rpm) for 30 min. PIC was radiolabeled at a ratio of 4 MBq ^111^In to 1 μg of PIC. To complex unbound ^111^In, ethylenediaminetetraacetic acid (EDTA) was added to a final concentration of 5 mM to. The labeling efficiency was determined using instant thin-layer chromatography on silica gels chromatography strips (ITLC-SG; Agilent Technologies) using 0.1 M sodium citrate buffer, pH 6, as mobile phase. Before in vivo administration, radiolabeled IFs were diluted with PBS (pH 7.4) to adjust to a volume of 200 μL/animal.

### Cell Lines

The murine melanoma cell line B16-OVA was cultured in RPMI 1640 supplemented with 10% FBS, 2 mM l-glutamine and 0.5% A/A, 1 mg/mL Geneticin (Gibco, 11811064), and 60 μg/mL Hygromycin B (Gibco, 10687010). Cells were split at a confluency of 70–80% by washing in 1× PBS followed by incubation until detachment in Trypsin-EDTA (BD Biosciences, 215240). Cells were incubated at 37 °C under 5% CO_2,_ humidified atmosphere.

### Mice

Mice were housed at the Central Animal Laboratory (Nijmegen, The Netherlands) in accordance with European legislation. All of the conducted protocols were approved by the local and national authorities (CCD, The Hague, The Netherlands; license numbers 10300-2015-0019 and 10300-2019-0020) for the care and use of animals with related codes of practice. Mice were female and between 6 and 10 weeks old at the start of the experiment. Mice were housed in IVC blueline or greenline cages with a maximal number of 6 mice per cage and were provided with ad libitum food and water and cage enrichment.

Animal studies at Oxford University (for the experiments with IF-A2^(NY-ESO-V)^ and IF-A2^(NY-ESO-V)^/IL-2) were conducted in accordance with the approval of the United Kingdom Home Office. All procedures were done under the authority of the appropriate personal and project licenses issued by the United Kingdom Home Office license number PBA43A2E4. In all experiments, mice were randomized to treatment and outcome assessment was blinded.

### Cell Culture of Primary Murine CD8^+^ T Cells

Murine OT-I CD8^+^ T cells or 1G4 CD8^+^ T cells were isolated from the spleen and lymph nodes of OT-I mice (C57BL/6-Tg(TcraTcrb)1100Mjb/Crl (Charles River)) or A2Eso1G4 HHD mice, respectively. Spleens and lymph nodes were digested with 20 ug/mL DNase I (Roche, 11284932001) and 1 mg/mL collagenase III (Worthington, LS004182) for at least 30 min at 37 °C. Organs were meshed through a 100 μm cell strainer, and red blood cells were lysed with in-house ammonium-chloride-potassium (ACK) buffer. CD8^+^ T cells were isolated with negative selection using the CD8a^+^ T Cell Isolation Kit, mouse (Miltenyi Biotec, 130-104-075) following manufacturer’s instructions. Cells were counted and diluted to desired concentration and cultured in RPMI 1640 (Gibco, 42401042) with 10% FBS, 2 mM L-Glutamine (Lonza Biowhittaker, BE17–605E/U1), 0.5% Antibiotic/antimycotic (Gibco, 15240-062) and 50 μM β-mercaptoethanol (Gibco, 21985023) in a 96 well U-bottom plate at 37 °C and 5% CO2. Cells used for proliferation studies were stained with 2.5 μM CellTrace Violet (CTV, Invitrogen, C34557) in 1% FBS in 1xPBS for 10 min at 37 °C and subsequent recovery for 30 min in 50% FBS at 37 °C. Cells were washed in 1xPBS and diluted to desired concentration in cell culture medium. Cells used for memory phenotype studies and Ki67 expression were cultured in a 48-well plate in 0.5 mL of culture medium supplemented with 300 IU/mL recombinant human Interleukin-2 (Proleukin, Novartis). Cells were split when necessary, and medium was replaced every 2–3 days. Murine 1G4 CD8^+^ T cells were processed as described above, except in that cells were frozen and thawed before functional assays. Cells were isolated from A2Eso1G4 HHD recipient mice, that were generated as described previously.^26^

### Ex Vivo CD8^+^ T Cell Activation Assays

IFs were diluted in cell culture medium to the desired concentration (see Table 1) and subsequently added to the culture medium. Cells were incubated with IFs until a specific time point was reached according to the experimental setup. Intracellular Granzyme B levels were assessed after 16 h of stimulation, followed by 5.5 h block of degranulation with 10 μg/mL Brefeldin A (Cayman Chemical, 11861-25) and 1:1000 monensin (eBioscience, 00-4505-51). In some experiments, Dynabeads Mouse T-Activator CD3/CD28 (11452D, ThermoFisher) in a 1:1 bead:cell ratio were added. Cell supernatant was taken at indicated time points and stored at −20 °C. Supernatant of murine 1G4 cells was taken on day 2. For wash off experiments, cells were incubated for either 30 or 90 min with IFs or free pMHC until the cells were transferred under sterile conditions to a 96-well V-bottom plate, washed 2× in 1× PBS and resuspended in cell culture medium. As a control, cells were left untouched until further processing. Supernatant was taken on day 2 and replaced with new cell culture medium, and proliferation was assessed on day 3.

### Ex Vivo Cytotoxicity Assay

B16-OVA cells were treated with 100 ng/mL murine IFNγ (Peprotech, 315-05) overnight. The next day, cells were harvested and stained with CellTrace Violet for some assays. Next, 10 000 B16-OVA cells were added to 96-well U-bottom plates and left to attach for 2–3 h. CD8^+^ T cells were stimulated for around 20 h and harvested using PBS with 2 mM UltraPure 0.5 M EDTA, pH 8.0 (Invitrogen, 15575-020), and the desired number was added in 100 μL of cell culture medium with 50 μM β-mercaptoethanol to the B16-OVA cells. As a control for background B16-OVA cell death, no T cells were added to the culture. After an additional 24 h, cells were harvested by taking off the medium, washing in 1× PBS and incubating with 30 μL of Trypsin-EDTA until all cells were detached. Cells were processed by flow cytometry. The percentage lysis was calculated using the following formula: % lysis = (1 – (Freq. treated viable target cells/Freq. no T cell viable target cells)) × 100.

### Antibodies and Reagents in Flow Cytometry

For flow cytometric analysis, cells were stained for 20–30 min at 4 °C in the dark using 1:2000 eFluor 780 fixable viability dye (eBiosciences, 65-0865-014), Propidium Iodide (Miltenyi Biotec, 130-093-233) or 1:2000 7-AAD (eBiosciences, 00-6993-50). Next, cells were stained for 20–30 min with a antibody mixtures. Murine OT-I CD8^+^ T cells of ex vivo assays were stained with aCD8a-PE (BD Biosciences, 553033), aCD8a-APC (Biolegend, 100712), aCD25-APC (eBiosciences, 17-0251-82), and aCD69-BV510 (Biolegend, 104531). Intracellular Granzyme B production was assessed by fixing the cells with BDCytofix/Cytoperm (BD Biosciences, 554714) for 20 min followed by incubation with aGranzymeB-PerCp/Cy5.5 (372211, Biolegend) for 30 min. Ki67 expression was assessed by fixing the cells for 60 min in Fixation/Permeabilization solution using the eBioscience Foxp3/Transcription Factor Staining Buffer Set (00-5523-00, invitrogen), followed by 2x washing for 5 min with the Permeabilization buffer provided in the kit. Next, cells were stained for 30 min with aKi67-PE (652403, Biolegend). The 1G4 murine CD8^+^ T cells were additionally stained with: mouse anti-human TCR Vβ 13.1-PE (clone: H131, Biolegend, 362409). For in vivo studies, cells isolated from selected organs were stained with aCD11b-PE (Biolegend, 101208), aCD45.1-PerCpCy5.5 (Biolegend, 110726), CD3-APC-Cy7 (Biolegend, 100221), aCD8-PE-Cy7 (Biolegend, 100722), and αCD16-αCD32 FcR block (BD Biosciences, 553142). Next, cells were washed 2× in PBA and transferred to polystyrene flow cytometry tubes. In cases where 7-AAD or PI was used, samples were stained 10 min at 4 °C before run in 1× PBS. Human transfected HLA-A2.1^+^ CD8^+^ T cells were stained with: anti-mouse TCR-β-FITC (Biolegend, 109205), anti-mouse TCR-β-BV421 (Biolegend, 109230), aCD25-PE-Cy7 (Biolegend, 302612), aCD8-BV510 (Biolegend, 344732). Samples were run on the BD FACSVerse (BD Biosciences) and analyzed using FlowJo vX0.7 after compensation using AbC Total Antibody Compensation Bead Kit (Invitrogen, A10497). Cell count was determined using Precision Count Beads (Biolegend, 424902). The division index was determined by manually gating on CTV peaks and using the following formula:  (where *i* is the division cycle and *N* is the proportion of cells in this division cycle.)

### Cytokine Levels in Supernatant

Cytokine levels were measured in cell supernatant using an enzyme linked immunosorbent assay (ELISA). Murine IFNγ levels and IL-2 levels were measured using the Mouse Interferon γ (IFNγ) Uncoated ELISA Kit (Thermo Fisher Scientific, 88-7314-76) or IL-2 Mouse Uncoated ELISA Kit (Thermo Fisher Scientific, 88-7024-76) following the manufacturer’s instructions. The supernatants of human CD19 CAR T cells or human TCR transfected CD8^+^ T cells were analyzed by IFNγ Human Uncoated Elisa Kit (Invitrogen, Thermo Fisher Scientific # 88-7316-88). Plates were read on a BioRad plate reader at a wavelength 450 nm and subtracted by 595 nm.

### Generation and Culture of Flt3 Ligand Dendritic Cells for RNAseq

Legs of C57BL/6 mice (Charles River) were collected. Bone marrow was washed out onto a 100 μm cell strainer, and cells were washed and plated in 10 cm Petri dish (Greiner, 633185) in supplemented cell culture medium as described above. The RPMI medium was additionally supplemented with 200 ng/mL human Flt3 ligand (Miltenyi Biotec, 130-096-479) and 5 ng/mL murine GM-CSF (Peprotech, 315-02) and 50 μM β-mercaptoethanol. Cells were cultured at a density of 15 × 10^6^ per plate at 10% CO2, 37 °C in humidified atmosphere. After 5 days, medium was replaced with fresh 200 ng/mL Flt3 ligand and 5 ng/mL murine GM-CSF. After 9 days, cells were harvested and replated at a density of 3 × 10^6^ cells per plate, and 200 ng/mL fresh Flt3 ligand and 5 ng/mL GM-CSF containing medium was added up to 10 mL. One day before T cell coculture, cells were stimulated with 0.3 μg/mL LPS (Invivogen, vac-3pelps). On the day of T cell coculture, cells were given 100 ng/mL SIITFEKL peptide (Genscript) for 3 h.

### Bulk RNAseq Experiment

For RNA sequencing experiments, OT-I CD8^+^ cells were incubated for 8 and 22 h with 5 ng of IF-pMHC^(SIIT)^/IL-2 or SIITFEKL-pulsed Flt3L DCs in a ratio of five OT-I CD8^+^ cells to two DCs in 37 °C, 5% CO2, humidified atmosphere. Viable cells were sorted on the BD FACSMelody into polypropylene tubes based on CD8-APC (Biolegend, 100721), CD103-PE (Biolegend, 121405) expression. Cells were lysed in 200 μL TRIzol (Thermo Fisher, 15596026) and stored at −80 °C before sending to Single Cell Discoveries B.V. (Utrecht, The Netherlands) for RNA extraction and bulk RNA sequencing. All samples passed the quality control with high quantity and quality, as assessed by Agilent Bioanalyzer. Sequencing was performed using an adapted version of the CEL-seq protocol. In brief: Total RNA was extracted using the standard TRIzol protocol and used for library preparation and sequencing. mRNA was processed as described previously, following an adapted version of the single-cell mRNA seq protocol of CEL-Seq.^46,47^ In brief, samples were barcoded with CEL-seq primers during a reverse transcription and pooled after second strand synthesis. The resulting cDNA was amplified by an overnight in vitro transcription reaction. From this amplified RNA, sequencing libraries were prepared with Illumina Truseq small RNA primers. The DNA library was paired-end sequenced on an Illumina Nextseq 500, high output, with a 1 × 75 bp Illumina kit (R1: 26 cycles, index read: 6 cycles, R2: 60 cycles). For data analysis, Read 1 was used to identify the Illumina library index and CEL-Seq sample barcode. Read 2 was aligned to the Mouse mm10 + mitochondrial genes reference transcriptome using BWA MEM.^48^ Reads that mapped equally well to multiple locations were discarded. Mapping and generation of count tables were done using the MapAndGo script1. A complete overview of the genes significantly different between DCs and IF-pMHC^(SIIT)^/IL-2 is provided in Table S1.

### Transfection of Human T Cells with TCR

CD8^+^ T cells were obtained using Ficoll density gradient centrifugation (Lymphoprep, ELITechGroup) followed by CD8^+^ T cell isolation (CD8^+^ T Cell Isolation Kit, human, Miltenyi Biotec) on buffy coats from HLA-A2.1^+^ human donors after written informed consent in accordance with the Declaration of Helsinki and in agreement with institutional guidelines. CD8^+^ T cells were transfected as previously described^49^ with mRNA (5 mg/mL, BioNTech RNA Pharmaceuticals) encoding for a murinized T cell receptor (specific for the HLA-A2.1-specific NY-ESO-1 epitope SLLWITQC). Transfection efficiency was determined 1 day after transfection using anti-mouse TCR-β-FITC (BioLegend, 109205) and was typically between 80 and 90%. Cells were stained with CTV as described above and treated with IF. Supernatant was taken 3 days after stimulation, and proliferation was evaluated 4 days after stimulation. For activation studies, cells were not stained with CTV and activation was read out one day after IF stimulation. Cells were cultured in an X-Vivo 15 (BE-02-060F, Lonza) with 2% human serum.

### Stimulation of CD19 CAR T Cells

Human CD3^+^ T cells were obtained from buffy coats of healthy donors by negative selection using a RosetteSep Human T Cell Enrichment Cocktail Kit (STEMCELL Technologies) and stored at −80 °C until use. T cells were cultured with X-VIVO 15 Cell Medium (Cultek, BE02-060Q) supplemented with 5% AB human serum (Sigma, H4522), penicillin-streptomycin (100 mg/mL), and IL-2 (50 IU/mL; Miltenyi Biotec), and stimulated with Dynabeads (Gibco, 11131D). After 24 h of T cell stimulation with beads, cells were transduced with anti-CD19 CAR lentiviral particles at a multiplicity of infection (MOI) of 5 and cultured for 6 days. Dynabeads were removed and supplemented medium was added to cells. T cell culture was maintained with X-vivo + 5% AB human serum + 100 mg/mL penicillin-streptomycin for a period of 48h. Then, the expression of anti-CD19 CAR was assessed by cell-based fluorescence using Allophycocyanin (APC) AffiniPure F(ab’)2 Fragment Goat Anti-Mouse IgG, F(ab’)2 Fragment Specific (Jackson Immunoresearch #115-136-072) before adding IF. The expression of anti-CD19 CAR on T cells in all experiments were ranging between 40 and 50%. Afterward, IFs were added to CD19 CAR T cell culture (1 × 10^6^ cells/mL) at a final concentration of 2 μg/mL. CD19 CAR T cells were split during the stimulation with IFs and supplemented X-VIVO without IL-2. The supernatant of the CD19 CAR T cells was collected at different time points (24 h, 72 h, and 5 days after adding IF).

### In Vivo Biodistribution and SPECT/CT Imaging

C57BL/6 mice received 290 ng of pMHC intravenously either bound or unbound to IFs or blank IFs labeled with indium-111. After 15 min, 1 h, 2 h, 4 h, 8 h, 24 h, 48 h, and 72 h, blood was collected from the mice through thigh bone puncture. After 24 or 72 h, mice were sacrificed by CO_2_ asphyxiation, and some mice were used for SPECT-CT analysis as described below. Organs were harvested from mice, and radioactivity was measured using a gamma counter (Wizard, PerkinElmer). The % of injected dose per gram of tissue/organs was calculated from the amount of radioactivity measured in aliquots of the injected dose. The %ID of lymph nodes was calculated using a similar formula but not normalized to weight. The %ID muscle was adjusted to the average weight of lymph nodes, which was calculated to be 3.5 mg. Of three randomly picked mice per group, CD11c^+^ (130-108-338, miltenyi), CD11b^+^ (130-049-601, miltenyi), and CD3^+^ (130-095-130, miltenyi) were isolated according to the manufacturer’s instructions and counted, and cellular uptake of ^111^In labeled IFs was measured using a gamma counter (Wizard, PerkinElmer). At 24 and 72 h after injection of ^111^In labeled IFs, one mouse of each group was imaged with SPECT/CT after euthanization. Images were acquired for 2 h with the U-SPECT-II/CT instrument (MILabs, Utrecht, The Netherlands) using a 1.0 mm diameter pinhole mouse high sensitivity collimator, followed by CT scan (615 μA, 65 kV) for anatomical reference. Scans were reconstructed with MILabs reconstruction software using a 16-subset expectation maximization algorithm, with a voxel size of 0.4 mm and 1 iteration. SPECT/CT scans were analyzed and maximum intensity projection (MIP) were created using Inveon Research Workplace software (Siemens).

### In Vivo CD8^+^ T Cell Proliferation

For proliferation studies with OT-I T cells, CD8^+^ OT-I T cells were isolated and labeled with CTV according to the aforementioned protocol. Depending on the experimental setup, 0.5 × 10^6^ or 1 × 10^6^ CTV labeled cells were injected intravenously. One day later, the amount as indicated in the figure legend was injected either iv or sc. After 3 or 4 days, spleen and lymph nodes were isolated and processed for flow cytometry. Samples were run on the MACS Quant or BD FACSVerse and gated for live cells.

For proliferation studies with 1G4 T cells, 1G4 T cells were isolated using “untouched isolation” MACS microbead selection (Miltenyi Biotec). Cells were labeled with CTV by mixing 1:1 in PBS, cells at 2 × 10^7^ /mL with CTV at 5 μg/mL (Thermo Fisher), and incubating for 15 min at 37 °C followed by blocking in FBS and washing with complete medium according to the aforementioned protocol. Depending on the experimental setup, 1 × 10^6^ CTV labeled cells were injected intravenously. One day later, the amount as indicated in the figure legend was injected intravenously. After 3 or 4 days, spleen and lymph nodes were isolated and processed for flow cytometry. Samples were run on the MACS Quant, BD FACSVerse or BD LSR Fortessa and gated for live cells.

### Lung Metastasis Model

C57BL/6 mice were injected iv with 0.25 × 10^6^ freshly isolated OT-I CD8^+^ T cells, preactivated OT-I CD8^+^ T or 1× PBS on day 0. The next day, mice received 1.4 μg of pMHC either bound to IFs or free pMHC or PBS iv After 4 days, 0.8 × 10^6^ B16-OVA cells, pretreated overnight with 100 ng/mL murine IFNγ, as described above, were added in 1× PBS iv. The weight and health of mice were followed throughout the study. After 14 days post tumor cell injection, mice were euthanized using CO_2_ asphyxiation, and lungs were perfused with cold PBS 2 mM EDTA through the right ventricle of the heart. The lungs were fixed in in-house Fekete’s solution, and lung metastases were counted the next day under blinded conditions. Metastatic burden on the lung surface was quantified by dividing the total metastatic area by total lung area using ImageJ Fiji software.^50^

To generate preactivated OT-I cells for adoptive transfer, freshly isolated OT-I CD8^+^ cells were cultured for 2 days in 24-well plates coated with 1 μg/mL anti-CD3 and 2 μg/mL anti-CD28 in culture medium. Next, cells were washed and cultured in new wells in cell culture medium supplemented with 300 IU/mL IL-2 for 4 more days.

### Subcutaneous Tumor Model

C57BL/6 mice were injected sc with 100 μL of 0.25 × 10^6^ B16-OVA cells in matrigel. When tumors reached a size between 50 and 100 mm^3^, mice were randomized according to tumor volume, and 0.4 × 10^6^ OT-I T cells were adoptively transferred intravenously. One day later, 200 μg of InVivoMab rat anti-mouse PD-1 antibody (clone: RMP1-14, BioXCell, BE0146) was injected in InVivoPure pH 7.0 Dilution Buffer (BioXCell, IP0070) intraperitoneal in some mice, which was given again on days 4 and 7. One day after OT-I T cell transfer, mice either received 0.5 μg of pMHC^(SIIN)^ on IFs or blank IFs in similar amounts as to the IF-pMHC^(SIIN)^, diluted in sterile PBS sc around the tumor. The weight and health of mice was followed throughout the study. Tumor sizes were measured every other day with a caliper. Tumor volumes were calculated as follows: width × length × depth × 0.4. Tumor growth was followed, and mice were sacrificed when the tumor reached the ≥1500 mm^3^ threshold. For the representation of tumor growth graphs, tumor volumes of dead mice were kept at the threshold value after euthanization until the end of the experiment. Tumors were formalin-fixed (10% formalin) and paraffin-embedded (FFPE) for histological analysis.

### Immunohistochemistry

Slides of 4 μm FFPE B16-OVA tumors were cut. Next, slides were deparaffinized and antigens were retrieved using ENVISION Flex Target Retrieval solution (Dako Omnis, K8004) in a microwave (3 min 1000W, 20 min 180W). Endogenous peroxidase was blocked 10 min in 3% H_2_O_2_ (Merck, 107209) washed and then blocked in 1% bovine serum albumin in TBS-T for 10 min. Afterward, slides were incubated with rabbit anti-mouse CD8a (dilution: 1/1000, clone: EPR21769, Abcam, ab217344) for 1 h at room temperature. Secondary BrightVision poly-HRP anti-rabbit antibody (dilution: 1/2, ImmunoLogic, S/DPVR-HRP) was applied for 30 min at room temperature. Next, Envision Flex HRP Magenta Substrate Chromogene System (Dako Omnis, DM857) was applied for 5 min. The reaction was stopped by washing for 1 min in tap water. Between each step, slides were rinsed in TBS-T. Slides were counterstained in hematoxylin and enclosed with QuickD mounting solution (Klinipath, 7281).

### Tissue Imaging and Quantitative Digital Analysis

Whole tissue slides were imaged with the Vectra Intelligent Slide Analysis System (Version 3.0.4, PerkinElmer Inc.) as previously described.^51^ Phenochart (version 1.1.0, Akoya Biosciences) was used to select the tumor area for analysis. Spectral libraries were built based on unstained tissue, melanin pigmentation, and single staining consisting of hematoxylin for nuclear staining and EnvisionFlex magenta for CD8^+^ T cells. Training of the inForm Advanced Image Analysis Software (Version 2.4.8, Akoya Biosciences) was performed on a selection of 10 to 15 representative original multispectral images to discriminate between tumor and necrotic areas, cell segmentation, and phenotyping of CD8^+^ T cells, melanin-pigmented cells, and other cells (Figure S14). Batch analysis of multiple original multispectral images of the same tumor was allowed by saving settings applied to the training images within an algorithm. The numbers of intratumoral CD8^+^ T cells were quantified and normalized for the tumor area (cells/mm^2^).

### Statistics

All data is represented as mean ± standard error of the mean (SEM). Graphs were generated in GraphPad Prism (version 8.0.2) or R Studios (version 4.0.3). Statistical analysis was performed on transformed data where appropriate, using Graph Pad Prism with the appropriate testing methods as indicated in the figure legends. Statistical significance was defined as a two-sided significance level of < 0.05. Only *p*-values < 0.05 are indicated in the graphs.

## Data Availability

The RNA sequencing is available in the GEO database (GSE215208). An overview of the normalized counts of all detected genes and of significantly different genes after 8 and 22 h can be found in Table S1. The rest of the data is available from the authors upon reasonable request.

## Supporting Information Available

Normalized counts by RNAseq for all detected genes and of significantly different genes between between DCs and IF-pMHC^(SIIT)^/IL-2 (XLSX)
Polymer characterization, OT-I T cell activation and target cell killing, OT-I T cell granzyme production and effector memory phenotype, OT-I T cell viability and proliferation in response to IF-pMHC^(SIIT)^ and IF-pMHC^(SIIT)^/IL-2, differential genes of OT-I T cells after stimulation with DCs and IF, antigen-specific stimulation of TCR-transfected T cells, CAR T cell activation, in vivo cellular uptake of IF, in vivo biodistribution and T cell activation of IF-pMHC^(SIIN)^, in vivo OT-I T cell expansion, in vivo 1G4 T cell expansion, enumeration of lung metastasis and mouse weight in iv B16-OVA tumor model, tumor growth and T cell infiltration in sc B16-OVA tumor model, image analysis approach of T cell infiltration into tumors, normalized counts of RNAseq (PDF)
